# Teaching statistics using dance and movement

**DOI:** 10.3389/fpsyg.2015.00050

**Published:** 2015-02-02

**Authors:** Lucy T. Irving

**Affiliations:** ^1^Imperial Horizons, Centre for Co-Curricular Studies, Imperial College LondonLondon, UK; ^2^Department of Psychological Sciences, Birkbeck, University of LondonLondon, UK; ^3^Department of Psychology, Middlesex UniversityHendon, London, UK

**Keywords:** dance, teaching, statistics, embodied learning, public engagement

A BPS Public Engagement Grant was awarded to the author, with Dr. Carl Senior of Aston University, to make a series of films which helped to demonstrate statistical concepts in psychology using dance and movement. These films have engaged students and educators internationally and across many sectors, not only the intended psychology students, with over 100,000 collective views on YouTube. Many educators have remarked upon how they could use them in their own classes. This paper reviews the anecdotal and empirical evidence in this area and invites others to contribute to a discussion of artistic ways of engaging students, both at university and more generally.

The response to these pieces has been surprising, not least because the films have been so far-reaching and apparently useful for so many with an interest in statistics. Blogs and articles by educators show that students of all ages and from a wide range of sectors have been introduced to the concepts, from nursery school children who were encouraged to describe and compare the dances in very basic terms, to trainee veterinary surgeons, marketing students, dancers studying business, nurses, as well as the expected psychology and social science students. Exactly what it is about the films that makes the concepts more lucid is unclear and some research in this area would be welcome. In his “Dance Lab” at the University of Hertfordshire, Dr. Peter Lovatt has conducted research into the effects of participating in and watching dance. However, little empirical literature has been published which looks at dance as a pedagogical tool. A small amount of literature, as well as anecdotal evidence suggests that learning in this way works, at least for some. But why? Stolberg (2006) discusses a handful of educators who have successfully communicated science through dance and presents some evidence supporting pedagogies which include collaborations between dance and science (see also Hanna, 2001). Keinanen et al. (2000) looked at studies which investigated whether “dance instruction” improved reading skill and found only small effect sizes. However, McMahon et al. (2003) found that a dance-integrated program improved basic reading comprehension in first-grade students in comparison to a control group. A number of schemes also exist in primary and secondary education which utilize movement and dance in the curriculum. In her dance classes at Brindishe Green School, Jenny Powel uses this “embodied learning” to get pupils thinking about topics in other areas of the curriculum, and research at The Place (“LearnPhysical,” Twiner et al., 2010) and the Open University (Grainger and Barnes, 2006) suggests that dance can improve cross-curricular learning (see also “Human Body, Reading and Tessellation” and “Dancing in Science” in the Great Primary Lesson Ideas series by The STEM Centre, n.d., ^1^, ^2^). One paper tested whether dance and movement improved understanding of electrocardiograms in third-year pharmacy students (Schultz and Brackbill, 2009). They observed improved student scores for those who participated in the dance condition. However, some said they were out of their comfort zone. This type of learning isn't for everyone.

“Statistics” and “dance” aren't words that are often uttered in the same sentence. However, this appears to be changing. The increasingly popular annual “Dance Your PhD” competition was started by John Bohannon who states in his TEDx Brussels talk entitled “Dance vs. Powerpoint, a modest proposal” (Bohannon, 2011), that “dance really can make science easier to understand,” and that sometimes “the ideal may be to use no words at all.” There are a number of organizations specifically dedicated to the integration of art with science and maths (for example, Maths Busking, The SciArt Center, Maths Dance, Art & Science Journal, Art of Science, Sci Art and Sci Arts), and there exist annual prizes for integration and collaboration in this area (for example, the Art Science Prize, artscienceprize.org). The popularity of these approaches suggests that there is an appetite for this kind of work. Many of these initiatives are designed for younger students, with little of this type of initiative available to students in higher education. This may not be surprising when one considers the often quite formal attitude that may be adopted at university. However, as Eric Stern and Karl Schaffer claim in their TED talk entitled “Math Dance” (TEDx Manhattan Beach, 2012) (Stern and Schaffer, 2012), using movement in the classroom works and is far from a distraction. Their interest is the connection between ideas and movement, and they state that “embodying the problem is memorable, social, creative… it makes mathematical ideas accessible,” and this is certainly supported by the anecdotal evidence surrounding the BPS Dancing Statistics films. Of course, many lecturers already incorporate embodied learning in their work. There's the famous “did you see the gorilla?” experiment demonstrating selective attention which is often re-enacted in cognitive psychology lectures, or the colleague who had a faculty member don a disguise and “break in” to the main lecture theater, mid-lecture, run down the stairs between all 200 students, cut the lecturer's tie, throw a custard pie in his face before escaping in an attempt to demonstrate the problems surrounding eye-witness testimony, and the lecturer who requests student volunteers to help demonstrate fMRI machines by spinning around at the front of the lecture hall. What seems to work about these examples is that they are all physical, are often funny, and are memorable to students; there was some action, their mates were involved, it was something different and it was unexpected.

Demonstrating complex statistics using dance is not to “sugar coat” the concepts; the films are not substitutes for lectures. Rather, the aim is to engage students and make them think about statistical concepts in different and memorable ways. Potentially this could take away some of the fear many psychology undergraduates experience when faced with this new way of thinking; there is a saying *it's like learning Greek* when faced with mastering a challenging topic. In this case it literally is. And there's some algebra thrown in there too for good measure. There may even be three or four names which describe the same thing, depending on current trends, whether you're reading a US or UK text, etcetera etcetera. What a minefield. Is it any wonder psychology students are often afraid of the mere idea of “statistics” before they even begin?

One does not passively watch these films. Rather, the audience is gently guided through each film and told what to consider as they watch. This brings an interactive feel to the pieces, something students often say is important for their engagement on a course. Being featured on the BPS YouTube channel means people can watch them anytime on computers, smart-phones and tablets and means they can view them anywhere with an internet connection. Students like the flexibility to study in their own way, at times that suit them and on their own devices (any lecturer knows how difficult it is to get students to use the university email address rather than their Hotmail or Gmail ones). Designing teaching methods which acknowledge this pedagogical development is crucial if we want to continue to recruit dynamic and enthusiastic students as we may lose them if we don't. An increasing number of faculty members working in higher education are using social media in their classrooms, with videos from YouTube (or elsewhere) being rated as the most valued way of using social media for teaching (Moran et al., 2011). Gone are the days of packed lecture theaters and 5 days per week spent on campus. Much more common now are distance learning, “webinars,” and virtual learning environments, as well as the use of technology in the classroom (Berk, 2009), a development undoubtedly influenced by the advancement of “Web 2.0” (the increasingly collaborative and social nature of the Internet, O'Reilly, 2005). Berk (2009) suggests that benefits to learning from using video in teaching may include generating interest; creating a sense of anticipation; increasing imagination; increasing recall of content and flow of ideas; being inspiring and motivating; making learning fun, and, perhaps most relevant here, “decreasing anxiety and tension on scary topics” (p. 2). Easily available online resources are increasingly valuable for this climate. YouTube may be one of the first places students go to when they need the answer to a problem (Duffy, 2008).

There are no voiceovers on the Dancing Statistics films. This was decided initially to reduce the risk of them sounding like mini-lectures. However, it has proved doubly-fortuitous as it means they can be understood without sound. This is beneficial for both students and lecturers as in the latter case they can turn down the volume and provide their own commentary. One of the most pleasing pieces of feedback received was the films being described as “little meditations: one has to concentrate, but not that hard” (personal communication, 2013). The YouTube comments and “Tweets” about the films are overwhelmingly positive, with a sizeable number of them referring to the fact they explain “stats without numbers.” In the year since their release, I have had requests for the captions in the films to be translated into Hebrew, French, and Spanish for use in universities in Israel, Canada and Costa Rica.

A project in the United States, which is receiving increasing amounts of attention, aims to engage schoolchildren in urban areas who are “under-achieving” (Emdin, 2010). The teaching revolves around hip-hop music and culture, a salient and prevalent influence in many of the students' lives. It is clear that the purpose is to engage students by connecting with them at a level which they are comfortable with. As Emdin states of students learning science, “it must be clear that disinterest, lack of participation, or poor performance is not the result of an intellectual deficiency, or an inability to grasp the content. It is rooted in an inability of educators to teach a new way” (2010, p. 10). A similar claim could be made for the teaching and learning of statistics. This approach is certainly not about being cool and “down with the kids,” something that's usually embarrassing for all involved. Rather, it is acknowledging what students can relate to and using that connection to spark their interest and engage them in a new way.

## Conflict of interest statement

The author declares that the research was conducted in the absence of any commercial or financial relationships that could be construed as a potential conflict of interest.

## Footnotes

^1^National STEM Centre. Human Body, Reading and Tessellation, from the Great Primary Lesson Ideas series, Playback Schools., (n.d.). [Video file]. Retrieved December 17th, 2014 from http://www.playbackschools.org.uk/programme/2015/human-body-reading-and-tessellationdy-reading-and-tessellation

^2^National STEM Centre. Dancing in Science, from the Great Primary Lesson Ideas series, Playback Schools., (n.d.). [Video file]. Retrieved December 17th, 2014 from http://www.playbackschools.org.uk/programme/1351/dancing-in-science
